# Genome-Wide Signatures of Selection Reveal Genes Associated With Performance in American Quarter Horse Subpopulations

**DOI:** 10.3389/fgene.2018.00249

**Published:** 2018-07-19

**Authors:** Felipe Avila, James R. Mickelson, Robert J. Schaefer, Molly E. McCue

**Affiliations:** ^1^Department of Veterinary Population Medicine, College of Veterinary Medicine, University of Minnesota, St. Paul, MN, United States; ^2^Department of Veterinary and Biomedical Sciences, College of Veterinary Medicine, University of Minnesota, St. Paul, MN, United States

**Keywords:** selection signatures, SNP, genotyping, horse, ancestral haplotypes, imputation

## Abstract

Selective breeding for athletic performance in various disciplines has resulted in population stratification within the American Quarter Horse (QH) breed. The goals of this study were to utilize high density genotype data to: (1) identify genomic regions undergoing positive selection within and among QH subpopulations; (2) investigate haplotype structure within each QH subpopulation; and (3) identify candidate genes within genomic regions of interest (ROI), as well as biological pathways, predicted to play a role in elite performance in each group. For that, 65K SNP genotyping data on 143 elite individuals from 6 QH subpopulations (cutting, halter, racing, reining, western pleasure, and working cow) were imputed to 2M SNPs. Signatures of selection were identified using *F*_ST_-based (*d*_*i*_) and haplotype-based (hapFLK) analyses, accompanied by identification of local haplotype structure and sharing within subpopulations (hapQTL). Regions undergoing positive selection were identified on all 31 autosomes, and ROI on 2 chromosomes were identified by all 3 methods combined. Genes within each ROI were retrieved and used to identify pathways and genes that might contribute to performance in each subpopulation. These included, among others, candidate genes associated with skeletal muscle development, metabolism, and central nervous system development. This work improves our understanding of equine breed development, and provides breeders with a better understanding of how selective breeding impacts the performance of QH populations.

## Introduction

The American Quarter Horse is the most common horse breed in the United States, with approximately 2.5 million registered individuals (2016 AQHA Annual Report)^1^ representing one third of the country's equine population. When accounting for the international equine population, the worldwide number of Quarter Horses (QH) reaches close to 3 million registered individuals (2016 AQHA Annual Report). Formally recognized as a breed with the establishment of the American Quarter Horse Association (AQHA) in 1940, the QH is a versatile and rugged breed that has been increasingly bred for performance in various disciplines. This versatility translates into profitability for the US equine industry: in 2015, over 130 million dollars were paid in awards or purses in AQHA-approved shows and races (2015 AQHA Annual Report)^2^.

Previous studies have shown that genetic diversity within the QH is high when compared to other horse breeds (McCue et al., 2012; Petersen et al., 2013a,b), which can be attributed to factors including genetic influence from other breeds (such as the Thoroughbred), as well as rapid and continuous population expansion (Petersen et al., 2014). Petersen et al. (2014) used both pedigree analysis and SNP genotyping data to determine if genetic differences exist across the QH breed itself by examining elite individuals from 6 performance groups (cutting, halter, racing, reining, western pleasure, and working cow). This work demonstrated that, with the exception of the working cow and cutting horse subpopulations, each performance group constitutes a genetically distinct subpopulation, a result of increased inbreeding over the past 75 years. Moreover, these data demonstrated that the QH subpopulations are genetically distinct due to the use of different breeding programs and different sire lines among different performance groups (Petersen et al., 2014).

Selection for a trait, accomplished through selective breeding practices, results in increased frequency of haplotype(s) containing the gene(s) and functional allele(s) conferring that phenotype at a rate greater than that expected under the null model of neutral evolution. This concept, termed a “selective sweep,” was first proposed by Smith and Haigh (1974). Genomic studies aiming at the identification of signatures of selection for phenotypes of interest, particularly performance traits, are being conducted in many livestock and domestic animal species; for example, cattle (Druet et al., 2014; Gutiérrez-Gil et al., 2015; Boitard et al., 2016; Randhawa et al., 2016), pigs (Li et al., 2014; Yang et al., 2014; Ma et al., 2015), sheep (Moradi et al., 2012; Fariello et al., 2013, 2014; Kijas, 2014), cats (Montague et al., 2014; Bertolini et al., 2016), and dogs (Akey et al., 2010; Boyko et al., 2010; Axelsson et al., 2013; Cagan and Blass, 2016). These studies have typically used a single method, such as the fixation index (F_ST_), extended haplotype homozygosity (EHH) or runs of homozygosity (ROH), to identify from several to hundreds of loci potentially under positive selection. Although some studies were able to identify functional alleles that are potentially driving selective sweeps, the use of complementary approaches such as the composite selection signals (CSS) method to aid in corroboration and prioritization of the identified ROI has been suggested (Randhawa et al., 2014, 2015).

Signatures of selection have also been identified in the horse genome. Petersen et al. (2013b) used Equine SNP50 BeadChip (Illumina, San Diego, CA, USA) data on 744 horses from 33 breeds, coupled with *d*_*i*_, a modified F_ST_ statistic (Akey et al., 2010), to identify and confirm loci associated with skeletal muscle fiber types in the QH (ECA18, *MSTN* gene), ability to perform alternative gaits in many breeds (ECA23, *DMRT3* gene), and size in draft breeds and the Miniature horse (ECA11:23.4Mb). Numerous other highly significant ROI identified in that study require further investigation to define candidate genes and propose putative functional alleles. Frischknecht et al. (2016) identified a selection signature on ECA1, containing the *IFF1R* and *ADAMTS17* genes, that is putatively associated with height in Shetland ponies, while Kader et al. (2015) reported genomic regions harboring candidate genes for height as undergoing positive selection in the Chinese Debao pony, including a region on ECA6 containing the height-associated *HMGA2* gene in many pony breeds (Frischknecht et al., 2015). Moon et al. (2015) collected whole genome sequence (WGS) data from Thoroughbred stallions and, using F_ST_ analysis between the Thoroughbred and the Jeju pony (an ancestral-type population), identified 12 regions putatively undergoing directional selection in the Thoroughbred. And, McCoy et al. (2014) found evidence of historical positive selection around the *GYS1* gene in Belgian horses, likely due to selection for enhanced glycogen deposition conferred by a gain of function mutation in glycogen synthase. Lastly, putative signatures of selection have been identified in racing and cutting QH subpopulations through relative extended haplotype homozygosity (REHH) analysis, combined with F_ST_ statistics (Meira et al., 2014; Beltrán et al., 2015). A total of 27 and 36 putative signatures of selection were identified in the racing and cutting lines, respectively, and putative candidate genes related to muscle, skeletal, cardiovascular, respiratory and nervous systems, vision, hearing, and cognition were identified (Meira et al., 2014; Beltrán et al., 2015).

In this study, we hypothesized that selective breeding within 6 QH subpopulations has increased the frequency of alleles responsible for elite performance, resulting in selective sweeps within each subpopulation. Based on this hypothesis, and relying on the principal of genetic hitchhiking, we used a combination of three approaches to identify genomic regions undergoing selection in elite QH performers that are likely to harbor alleles underlying performance traits. Within each of these regions, we identified genes and biological pathways predicted to play an important role in performance, which warrant further investigation to identify the underlying functional alleles.

## Materials and methods

### Sample collection and genotyping

The individuals and Equine SNP70 genotyping data used in this study have been previously described (Petersen et al., 2014) and are publically available (animalgenome.org)^3^ Briefly, all horses included in this study were among the 200 top performers of each of the 6 subpopulations (cutting, halter, racing, reining, western pleasure, and working cow) as determined by money or points earned in 2009 and 2010 according to the AQHA, after eliminating full and half-sibships. Primary performance characteristics of these populations are provided in Table 1.

**Table 1:** Primary characteristics of the six QH performance groups evaluated in this study, as described by the American Quarter Horse Association.

**Performance Type**	**Characteristics**
Cutting	Horse and rider must move quietly into a herd of cattle, cut one cow from the herd, drive it to the center of the arena and “hold” it away from the herd. The horse is scored on its ability to keep the cow from returning to the herd, cow sense, attentiveness, and courage.
Halter	Horses are led before judges so that lameness and quality of movement can be evaluated. Horses are judged on conformation including: balance, structural correctness, breed, sex characteristics and degree of muscling.
Racing	Horses race against one another at distances between 220 and 870 yards. The classic distance is 440 yards (1/4 mile).
Reining	The horse is judged on movement under saddle, mastery of a prescribed maneuver and attitude as it is guided through a specific pattern. The horse is required to perform stops, spins, rollbacks, lead changes and circles at a lope.
Western pleasure	Horses are evaluated on quality of movement under saddle at the walk, jog, and lope, while staying quiet, calm, and traveling on a loose rein.
Working cow (Reined cow horse)	Combines reining ability and cow sense. The competition consists of two parts: prescribed reined work and actual cow work. Judging is based on good manners, smoothness, cow sense and ease of reining.

*Originally provided in Petersen et al. (2014)*.

DNA was isolated from hair root samples of 143 elite individuals (24 individuals each from the cutting, halter, racing, reining, and working cow subpopulations, and 23 from the western pleasure group). These samples were genotyped on the Illumina Equine SNP70 BeadChip (Petersen et al., 2014) that contains approximately 65,000 SNP markers. For the current study, a subset of 47 individuals (16 from the halter, 15 from the racing, 9 from the western pleasure, and 7 from the working cow subpopulations), were also genotyped using the custom MNEc2M SNP array, which contains approximately 2 million SNPs (Schaefer et al., 2017).

### Genotype imputation

The Beagle 4.0 software (Browning and Browning, 2007) was used to impute the original 65,000 SNP genotyping data to approximately 2 million SNPs for all 143 individuals included in this study, on a per chromosome basis using default settings. Genotypes were imputed using a reference panel consisting of 1,934,984 SNPs and 485 individuals from 24 different breeds encompassing wide genetic diversity (Petersen et al., 2013a; Schaefer et al., 2017). The average genotype concordance across all chromosomes, measured by dividing the number of accurate calls (best-guess genotypes that matched the true genotype for a particular individual in the reference panel) by the total number of genotype calls, was 99.5% (Schaefer et al., 2017).

### SNP pruning

Because sex chromosomes are subjected to different selective pressures, have different effective population sizes and undergo more drift than autosomes (Heyer and Segurel, 2010), only autosomal SNPs were used in this study. After imputation, SNPs with genotyping call rates lower than 95% and minor allele frequency (MAF) of less than 0.05 across all individuals were removed (PLINK, Purcell et al., 2007). After quality control, a total of 1,928,388 SNPs remained for further analysis.

### Analysis using the *d_*i*_* statistic

Locus-specific divergence in allele frequencies for each QH subpopulation was calculated within non-overlapping 10 kb windows across the 31 autosomes using the *d*_*i*_ statistic, as previously described (Akey et al., 2010; Petersen et al., 2013b). For each genomic window, *d*_*i*_, or the locus-specific divergence of allele frequency for a given subpopulation compared to the genome-wide average of pair-wise F_ST_ values for that subpopulation relative to all other subpopulations (expected divergence), was computed (Schaefer, 2018). In other words, this statistic detects locus-specific deviation in allele frequencies for each subpopulation relative to the genome-wide average of pairwise F_ST_ values summed across all 6 subpopulations. This method was chosen for calculation because normalizing the standard F_ST_ statistic reduces the incidence of false positive tests that are driven by evolutionary relationships between populations rather than by selective pressures. Further, unlike single marker tests, the computing of F_ST_ across haplotype windows helps to control for stochastic variation and SNP ascertainment bias. The mean number of SNPs per 10kb window was 8.2 (± 3.2), and the total number of windows analyzed per subpopulation was 217,806. Significant *d*_*i*_ windows were considered as those corresponding to the top 0.1% of the empirical distribution (approximately 218 windows per subpopulation) and were considered putative signatures of selection. Two or more significant *d*_*i*_ windows located within 500 kb of each other were considered as a single ROI for analysis purposes.

### Analysis using hapFLK

The hapFLK is a haplotype-based approach applied to unphased genotypic data. The hapFLK 1.3.0 program version (https://forge-dga.jouy.inra.fr/projects/hapflk) was used to detect signatures of selection through haplotype differentiation among hierarchically structured populations, as described by Fariello et al. (2013). Briefly, FLK is an extension of the Lewontin and Krakauer (LK) test for the heterogeneity of the inbreeding coefficient, which uses phylogenetically estimated relationships between the subpopulations, overcoming limitations to the LK test due to highly correlated allele frequencies between subpopulations. HapFLK uses local haplotype data, the differences in haplotype allele frequencies between populations, and the hierarchical structure of subpopulations to identify genomic regions undergoing selection. The software was run on a per chromosome basis using a kinship matrix, constructed from population-based Reynolds distances, and the genotypes (^*^.PED and ^*^.MAP files) for each population. For this study, no outgroups were defined, 10 clusters (-K 10) were used for the fastPHASE model, and the hapFLK statistic was computed for 20 EM runs to fit the LD model (–nfit = 20). Then, *p*-values were computed for each SNP-specific value using a Python script provided with the hapFLK program, and values were considered significant ROI if –log_10_ (*p*-value) > 4, as applied previously (Fariello et al., 2013).

### Analysis using hapQTL

Local haplotype sharing within each subpopulation was calculated with hapQTL (http://www.haplotype.org) using default settings (Xu and Guan, 2014). This approach relies on a statistical model for linkage disequilibrium (LD) to infer ancestral haplotypes and their frequencies at each SNP marker for each individual within a population. In hapQTL, each SNP is used as a core marker to calculate the extent of local haplotype sharing (LHS)—or the probability of two individuals descending from the same ancestral haplotypes—within each subpopulation. For each analysis 1 EM run was used with 50 steps (-w 50), 3 upper clusters (-C 3), 10 lower clusters (-c 10), and with a prior LD length of 0.5 centimorgan (-mg 200). Clusters of SNPs with –log_10_ (Bayes Factor) > 5 were considered significant ROI, and orphan signals were removed from the analysis due to the fact that these do not constitute haplotypes.

### Candidate gene identification and prioritization

Candidate genes within the ROI identified by *d*_*i*_ and hapFLK were retrieved with the Ensembl BioMart tool (www.ensembl.org), using the EquCab2 dataset for each subpopulation. To refine the list of candidate genes in ROI and further understand their association with phenotypes of interest for each subpopulation based on their biological features, functionalities, and biological pathways, a list of seed genes based on hypothesized tissue and metabolic pathways of interest was generated using the PolySearch2.0 algorithm (Liu et al., 2015). The resultant list of seed genes was retrieved from PolySearch2.0 for skeletal muscle development, metabolism, and nervous system development (Supplementary Table 1). This list was further curated manually by analyzing relevant literature using PubMed (https://www.ncbi.nlm.nih.gov/pubmed) for candidate genes associated with phenotypes or pathways of interest for each subpopulation. The number of seed genes chosen for each phenotype was based on work described by Liu et al. (2015). Candidate gene prioritization within the ROI was then performed using Endeavor-GW (https://endeavour.esat.kuleuven.be/Endeavour.aspx) (Tranchevent et al., 2016).

### Pathway and functional analysis

Functional classification, statistical overrepresentation and pathway analyses of candidate genes for each subpopulation were conducted using PANTHER 11.1 (http://pantherdb.org/) (Mi et al., 2017), and FunRich3.0 (http://funrich.org/index.html) (Pathan et al., 2015), using default parameters. Specifically, fold enrichment analyses were included for: cellular component, molecular function, biological process, biological pathway, protein domain, site of expression, transcription factor, clinical phenotype associated with human homologs, their expression sites in humans, and results from the statistical overrepresentation analysis.

## Results

### Identifying signatures of selection using the *d_*i*_* statistic

**Figure 1:**
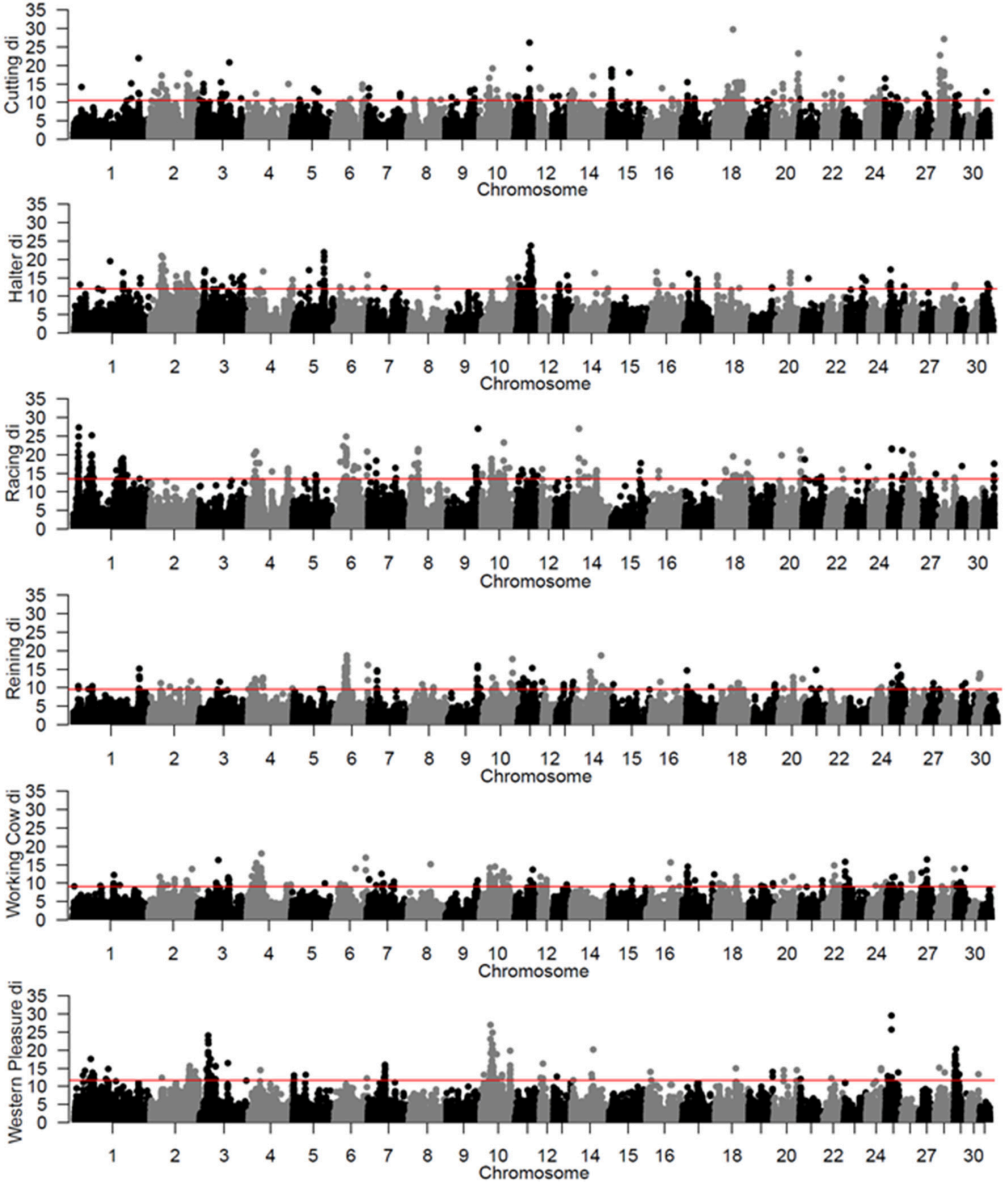
Genome-wide *d*_*i*_ values for the 6 QH subpopulations. Each *d*_*i*_ value is plotted on the y axis and each autosome is shown on the x axis in alternating colors. Each point represents a 10 kb window. The red horizontal line represents the top 0.1% of the empirical distribution of *d*_*i*_for each QH subpopulation.

To identify population-specific loci under positive selection in the 6 QH subpopulations, we calculated the *d*_*i*_ value (Akey et al., 2010; Petersen et al., 2014) for approximately 218,000 non-overlapping 10 kb windows along the genome (Figure 1). Each subpopulation had several clearly defined ROI comprising contiguous windows with significant *d*_*i*_ values, in addition to numerous ROI comprising a single or just a few windows. The number of ROI identified per subpopulation varied from 39 in the western pleasure subpopulation to 77 in the racing subpopulation, for a total of 346 ROI, with sizes ranging from 10 kb to 1.8 Mb (Table 2). The number of annotated genes located within these ROI ranged from 78 in the working cow subpopulation to 171 in the racing group, with an average of 105 genes identified per subpopulation. Across all 6 QH performance groups a total of 635 genes were situated within these putative signatures of selection. A complete list of the significant *d*_*i*_ windows (top 0.1% of the empirical distribution) for each subpopulation is provided in Supplementary Table 2.

**Table 2:** ROI identified by *d*_*i*_ analysis in the 6 QH subpopulations.

**QH subpopulation**	**Number of ROI identified**	**Number of genes**
Cutting	60	134
Halter	47	90
Racing	77	171
Reining	52	81
Western pleasure	39	81
Working cow	57	78
Total	346	635

*Totals do not consider overlap between populations or analysis methods*.

A total of 56 *d*_*i*_ windows were found to be shared between 2 or more subpopulations; of those, 50 were shared by 2 performance groups, 5 by 3 performance groups and 1 was shared by 4 performance groups (Table 3). The subpopulations with the most shared signatures of selection identified by *d*_*i*_statistics were racing and reining (14), and halter and reining (9). Interestingly, no significant *d*_*i*_ windows were shared between the racing and cutting subpopulations, nor between the racing and the western pleasure subpopulations. No *d*_*i*_windows were shared by all six subpopulations.

**Table 3:** ROI identified by *d*_*i*_ analysis that are shared by two or more QH subpopulations.

**Chromosome**	**Position (bp)**	**Cutting**	**Halter**	**Racing**	**Reining**	**Western pleasure**	**Working cow**
1	13,685,246			X	X		
1	119,564,500		X	X			
1	119,676,493		X	X			
1	161,715,511		X		X		
2	28,112,330		X		X		X
2	63,922,661		X				X
2	93,844,075		X		X		
2	93,904,657		X			X	
2	95,577,244					X	X
2	95,962,979		X			X	
2	96,425,441		X			X	
3	12,705,501	X	X				
3	68,445,127	X				X	
4	18,376,592				X		X
4	19,475,682			X			X
4	35,285,735		X		X		
4	106,747,371		X		X		X
5	65,517,082		X		X		
6	25,394,821			X	X		
6	26,913,211			X	X		
6	30,595,130			X	X		
6	30,637,559			X	X		
6	30,686,504			X	X		
6	30,705,641			X	X		
6	30,724,394			X	X		
6	30,753,624			X	X		
6	31,086,772			X			X
6	31,334,561			X	X		
6	81,474,997		X	X	X		X
6	81,534,461				X	X	X
7	5,529,473	X				X	
7	65,654,968			X			X
9	69,646,156			X	X		
9	70,735,787			X	X		
9	70,814,115	X			X		
10	23,574,776	X				X	X
10	23,956,861	X		X		X	
10	24,014,382					X	X
10	66,622,713			X	X		X
10	71,364,970		X			X	
10	83,744,000		X		X		
11	3,863,250	X	X				
11	35,553,211		X		X		
11	36,006,028		X		X		
11	38,335,749	X	X				
16	24,163,505			X	X		
17	15,344,171	X					X
18	19,864,538			X			X
18	50,904,645	X					X
20	12,885,496			X	X		
20	25,186,056					X	X
20	35,886,068		X		X		
25	6,263,862		X			X	
25	6,734,228			X			X
25	14,862,570				X	X	
28	14,573,111	X					X

### Identifying signatures of selection using hapFLK

**Figure 2:**
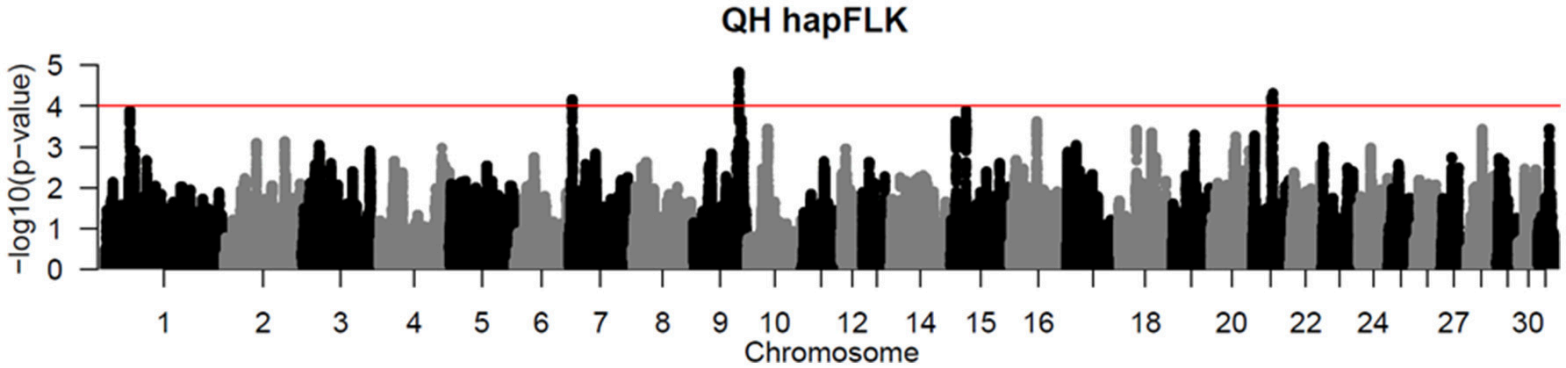
Haplotype-based hapFLK results for all 6 QH subpopulations. Chromosome number and statistical significance ([–log_10_] *p*-values) are plotted in the x and y axes, respectively. The genome-wide significance threshold corresponding to *P* < 0.0001 is shown as a horizontal red line.

Per chromosome hapFLK values were computed for all 6 subpopulations combined (total of 143 individuals), *p*-values were calculated from the null distribution of empirical values, and the negative log of *p*-values was plotted against the genomic position for each chromosome (Figure 2). A total of 6 ROI containing at least one significant peak [–log_10_ [*p*-value] > 4] in the hapFLK scan were identified on 5 different autosomes (Table 4). These regions, located on ECA1, 7, 9, 15, and 21 (with two nearby regions), harbor a total of 7 genes putatively undergoing selection.

**Table 4:** ROI identified by hapFLK and their overlap with ROI identified by *d*_*i*_ and hapQTL.

**Chromosome**	**Position (bp)**	**Lowest *p*-value**	**Gene symbols**	**Cutting**	**Halter**	**Racing**	**Reining**	**Western pleasure**	**Working cow**
1	40,502,663-40,641,889	0.000128973	*PTEN, ATAD1*	hapQTL	–	*d_*i*_*	hapQTL	–	–
7	5,501,423-5,596,628	0.000068200	*CERS4, CD320, RPS28*	Both	–	–	–	–	*d_*i*_*
9	70,643,295-70,822,536	0.0000146	–	Both	–	*d_*i*_*	Both	–	–
15	23,550,251-23,566,268	0.000127614	–	–	–	–	–	hapQTL	–
21	29,967,251-29,989,856	0.00006170	*PRLR*	hapQTL	hapQTL	hapQTL	hapQTL	–	–
21	31,684,806-31,731,726	0.00004790	*NPR3*	–	–	hapQTL	–	–	–

*Chromosomal location, genomic coordinates, lowest p-value and genes contained within each hapFLK ROI (left). For each QH subpopulation (right), “d_i_” denotes overlap between hapFLK and d_i_ hits; “hapQTL” denotes overlap between hapFLK and hapQTL hits; and “Both” indicates overlap between hapFLK, d_i_ and hapQTL*.

### Identifying haplotype structure using hapQTL

**Figure 3:**
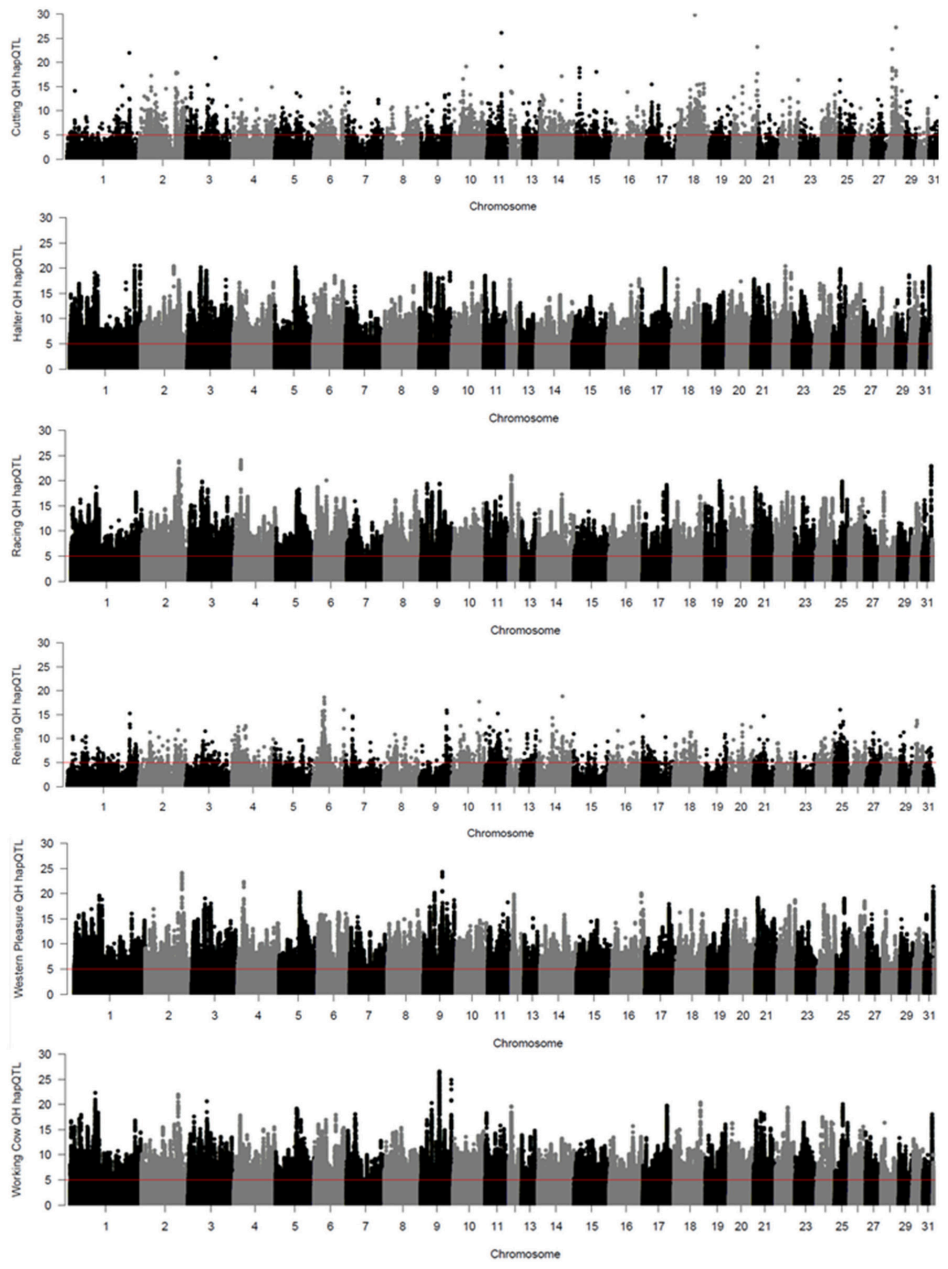
Genome-wide hapQTL values for the 6 QH subpopulations. Bayes factor values for each SNP are plotted on the y axis and each autosome is shown on the x axis in alternating colors.

Local haplotype sharing (LHS) within subpopulations was delineated by analyses using hapQTL (Xu and Guan, 2014). Clusters of contiguous significant SNP markers with –log (Bayes Factor) > 5 were considered as ROI as they most likely represent putative ancestral haplotypes shared by individuals from each subpopulation. Genome-wide hapQTL results for each QH subpopulation are shown in Figure 3, where shared ancestral haplotypes are shown as peaks containing contiguous significant SNPs. Each subpopulation displays numerous significant ROI, typically many on each chromosome, and spread across the entire genome. This approach was used to further validate the signatures of selection detected by the previously described methods, as such regions are most likely found in locally conserved haplotypes that are fixed or nearly fixed within each subpopulation. The number of significant SNPs varies widely across the 6 populations, from < 4,000 in the cutting and reining groups, to > 100,000 in the halter, racing, western pleasure, and working cow subpopulations. A complete list of all SNPs with significant hapQTL values for each subpopulation is provided in Supplementary Table 3.

### Summation and overlap among selection signature detection methods

**Figure 4:**
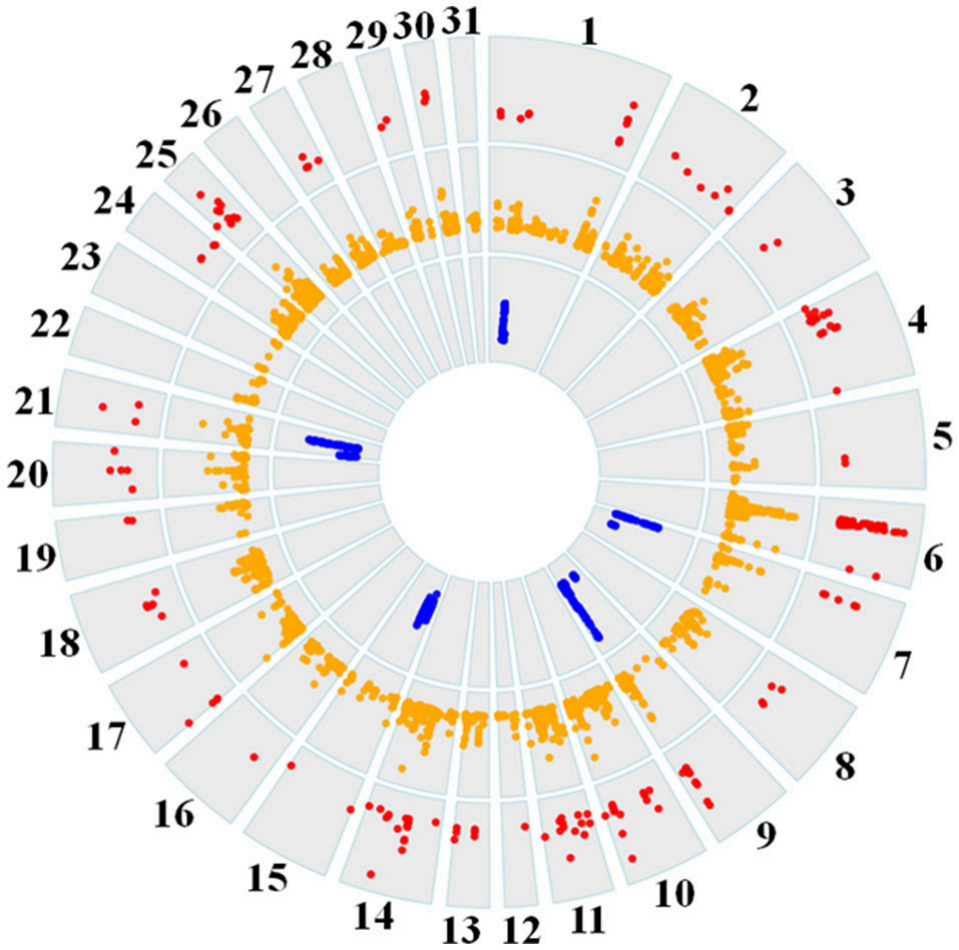
Circos plot showing genome-wide significant *d*_*i*_ values (red layer), hapQTL values (orange layer), and hapFLK values (blue layer) across all 31 autosomes for the reining QH subpopulation.

Genome-wide overlap in significant ROI identified by each of the three methods was assessed in each subpopulation (Figure 4; Supplementary Figures 1–6). Approximately 90% of significant *d*_*i*_ windows were contained within shared ancestral haplotypes identified by hapQTL, with little variation in the percent overlap between *d*_*i*_ and hapQTL ROI among the subpopulations. The observed overlap ranged from 178 shared hits (81% of significant *d*_*i*_ windows) in the reining subpopulation, to 206 common ROI (94% of significant *d*_*i*_ windows) in the racing subpopulation (Supplementary Figures 1–6). In addition, all 6 ROI identified by hapFLK were also found to be significant by the *d*_*i*_statistic, hapQTL or both methods (Table 4).

### Candidate gene prioritization

All annotated genes contained within ROI were retrieved using Ensembl BioMart (complete list provided in Supplementary Table 4). After retrieving annotated equine genes within ROI, PolySearch 2.0 (http://polysearch.ca) was used to generate a list of seed genes associated with traits of interest for improved performance (metabolism, skeletal muscle development, central nervous system). A manual curation of each list was performed using literature obtained from PubMed (https://www.ncbi.nlm.nih.gov/pubmed/) for their association with the aforementioned phenotypes. The resultant list of 33 seed genes: 11 using “skeletal muscle” as seed, 11 using “central nervous system” as seed, and 11 using “metabolism” as seed, is shown in Supplementary Table 1. These seed lists were used to prioritize candidate genes retrieved for each subpopulation based on phenotypes of interest using Endeavor-GW. Thus, in addition to the original seed genes, the output resulted in a total of 78–171 ranked genes for each subpopulation based on the phenotypes of interest: 635 across all subpopulations (602 unique, and 33 shared between 2 or more subpopulations) for use in pathway analysis (Supplementary Tables 5–7). Supplementary Table 8 lists the top 3 candidate genes per subpopulation, chosen according to possible associations with the desired performance characteristics based on their biological pathways and/or functions. Table 5 lists the 33 genes putatively undergoing selection in 2 or more subpopulations.

**Table 5:** Genes undergoing selection in 2 or more subpopulations.

**Gene Name**	**Cutting**	**Halter**	**Racing**	**Reining**	**Western pleasure**	**Working cow**
*ABCA13*				X		X
*ANKFN1*	X	X				X
*ARHGAP20*			X	X		
*ARMC12*		X		X		
*C11orf53*			X	X		
*CACNA2D1*		X				X
*COLCA2*			X	X		
*DCAF11*			X	X		
*DDX11*			X	X		
*DYNC1/2*	X					X
*FDX1*			X	X		
*GLE1*	X				X	
*GNE*	X			X		
*HECW1*			X	X		
*INVS*			X			X
*KIAA1191*	X				X	
*LENG8*					X	X
*LOXL1*		X	X			
*MAP3K20*			X			X
*PARD3*					X	X
*PIP4K2A*	X				X	X
*POU2AF1*			X	X		
*SLC25A27*	X					X
*SRPK1*		X		X		
*STOML1*		X	X			
*STX12*		X		X	X	X
*TBC1D21*		X	X			
*TPD52L1*				X	X	
*TSPAN9*			X	X		X
*TTYH1*	X				X	X
*UNC79*					X	X
*WASHC1*			X	X		
*ZPBP*			X			X

*Two genes (7SK and U6) were not counted because they have copies in several genomic loci*.

### Pathway and functional analysis

The biological functions and pathways in which genes from ROI are involved were assessed using PANTHER 11.1 (Mi et al., 2017) and FunRich3.0 (http://funrich.org/index.html) (Pathan et al., 2015). Supplementary Tables 9–14 provide PANTHER 11.1 and FunRich3.0 analysis reports for each QH subpopulation. These analyses provided information on the common features and shared functions of the genes putatively undergoing genomic selection in different QH performance groups, according to the expected performance profile of elite individuals in each category.

## Discussion

Our approach to detecting genome-wide signatures of selection in the QH subpopulations combines different methods to increase efficiency, reliability, and scope. This study revealed that there are hundreds of loci likely undergoing selection, of which many are almost certainly contributing to desired specialized phenotypes, in each subpopulation. Overlap between statistical methods to detect selection (namely *d*_*i*_ and hapFLK) assisted in pinpointing ROI that most likely constituted true signatures of selection within each subpopulation. Furthermore, the use of hapQTL to confirm that the vast majority of genomic regions undergoing selection constituted shared haplotypes within each subpopulation increased the robustness of our analysis. Although hapQTL was not designed specifically to identify genomic signatures of selection *per se*, it has been used to infer ancestral haplotype associations among individuals with similar phenotype (Xu and Guan, 2014). Therefore, combined with ROI identified by *d*_*i*_ and hapFLK, this approach allowed for a more confident interpretation of the association between candidate genes found within such ROI and the unique phenotypic features of each QH subpopulation. This work also reveals the many types of biological mechanisms that can contribute to the complex muscle, nervous system and metabolic phenotypes, as well as the difficulties likely inherent in attempting to identify the functional alleles associated with them.

Among the 27 signatures of selection identified by Meira et al. (2014) for the racing QH via REHH and F_ST_, a total of 14 overlap with the ROI identified by the present study: 6 of those were identified by *d*_*i*_ statistics, and 8 were highlighted by hapQTL. Of those, 4 were identified by both *d*_*i*_ statistics and hapQTL analysis. In addition, when comparing the 36 signatures of selection identified by Beltrán et al. (2015) in the cutting QH to those detected for this subpopulation in the present study, a total of 11 overlap: 2 identified by both *d*_*i*_and hapQTL, and 9 identified exclusively by hapQTL. A summary of these comparisons is shown in Supplementary Table 15. Overlap across studies for these ROI in these two subpopulations is encouraging, making them a top priority in attempting to identify the putative functional alleles associated with performance phenotypes. Nevertheless, our study also benefitted from a higher SNP density, utilizing different methods of detection, and the specific inclusion of only elite performers from 6 different QH subpopulations. This enabled us to define more, and likely highly important, signatures of selection. Moreover, our functional, network, and pathway analyses in the different populations show different populations of genes undergoing selection with different functions and interactions in each QH subpopulation. Several examples from each subpopulation are discussed below.

### The cutting subpopulation

Pathway analysis shows an approximate 30-fold enrichment in genes associated with formation of the pyruvate dehydrogenase (PDH) mitochondrial complex and with pyruvate metabolism (*DLAT, PDHX*; *p*-value = 0.001) in this subpopulation (Supplementary Table 9). Besides being involved in glucose metabolism, human studies suggest that variants in these genes are associated with neuromuscular disorders and neuronal function (Barnerias et al., 2010). Candidate gene prioritization for skeletal muscle development (Supplementary Table 5) and metabolism (Supplementary Table 7) confirms these findings by assigning high ranks for the aforementioned genes.

### The halter subpopulation

In the halter subpopulation, pathways associated with lipid metabolism and function, such as digestion of dietary lipid (59-fold enrichment; *p*-value = 0.016) and platelet sensitization by LDL (58-fold enrichment; *p*-value = 0.0004) were identified. It can be hypothesized that the selective breeding for lean and muscular phenotypes in this subpopulation may have led to the selection of genes involved in such metabolic pathways. On the other hand, we found no evidence for a signature of selection near the *SCN4A* gene locus on chromosome 11 (ECA11:15 Mb) in the halter horse subpopulation. It has been widely assumed that a combination of extensive breeding to a halter horse stallion carrying a *SCN4A* mutation responsible for the neuromuscular disorder hyperkalemic periodic paralysis (HYPP) (Rudolph et al., 1992), as well as resultant epistatic effects of this mutation on muscle contractility, was responsible for the perceived increase in muscling desired in this population. Our data suggests that *SCN4A* locus itself is not driving selection for the muscling in this halter horse line. On the other hand there is an ROI located 20 Mb upstream from *SCN4A* (ECA11:34–36 Mb) identified both by *d*_*i*_and hapQTL analyses, containing a total of 13 genes (Supplementary Table 4), that are potentially worth investigating for the presence of variants associated with skeletal muscle development in halter horses, such as *MYO19* (ECA11:35.7 Mb), associated with mitochondrial transport during mitosis.

### The racing subpopulation

Our *d*_*i*_ analysis identified four ROI on ECA1 (ECA1:12,256,704-14,342,963; ECA1:40,505,469-41,555,488; ECA1:45,034,220-46,901,028; ECA1:115,914,274-122,185,069) that are found exclusively in the racing QH subpopulation, were found to constitute ancestral shared haplotypes by hapQTL, and were not identified by Meira et al. (2014). Together, these “racing” ROI contain 99 genes, or approximately 58% of all genes undergoing selection in this subpopulation (see Supplementary Table 11). After prioritization, top candidate genes located in these and other loci showing association with the racing phenotype were also identified. One of the top candidate genes for racing ability is *BAG3* (BCL2-associated athanogene 3, ECA1:12.8 Mb); variants and/or deletions in this gene have been associated with myopathies, neuropathies and cardiomyopathies in various human subpopulations (Franaszczyk et al., 2014; Kostera-Pruszczyk et al., 2015), suggesting a potential involvement of this gene in skeletal muscle and heart function, essential phenotypes for individuals with increased racing ability. Another example is *PRKCA* (Protein kinase C alpha, ECA11:13.4 Mb), a member of the of serine- and threonine-specific protein kinase family. Knockout studies in mice suggest that this kinase may be a fundamental regulator of cardiac contractility and calcium handling in myocytes (Braz et al., 2004). Future studies of putative selection in Thoroughbreds and Standardbreds, breeds that have historically been selected for racing performance, will help validate and possibly expand this list of putative candidate genes for racing ability on ECA1 and elsewhere across the genome. Finally, pathway analysis showed a ten-fold enrichment in genes associated with netrin-1 signaling (*p*-value = 0.00054). These genes play an important role in axon guidance, on the structural integrity, and on the regulation of the transmission of the nervous stimulus through neuronal axons (Supplementary Table 7; Koticha et al., 2006). Regulation of such physiological functions is essential for improved athletic performance in racehorses.

### The reining subpopulation

Elite reining performers must possess skills that allow them to move effortlessly around the arena, quickly reacting to subtle hand and foot cues from the rider in order to execute movements such as loping, galloping, circling, spinning, and stopping. Therefore, reining horses must possess improved athleticism and intellectual abilities in order to excel in the sport. Analysis of genomic regions undergoing selection within this subpopulation reveals genes associated with such traits. Examples include *RDX* (ECA7:18.9 Mb), which plays a role in the binding of the barbed end of actin filaments to the plasma membrane and has been associated with skeletal muscle as well as behavior/neurological phenotypes in humans; *KIF1A* (ECA6:26.1 Mb), involved in axonal transport of synaptic vesicle precursors, and *BACE1* (ECA7:25.2 Mb), both associated with different neurological phenotypes in humans. It is worth noting that highly significant signals were detected using all 3 statistical approaches on a ~300 kb region of the genome in the reining QH subpopulation (ECA9:70.6 Mb−70.9 Mb; see Table 3, Supplementary Tables 2–3). Interestingly, this region does not contain any annotated genes in EquCab2, which might indicate that another genomic feature such as a regulatory sequence, non-coding RNA, or transposable element for example, might be driving selection in this region but cannot yet be identified due to the current state of the equine genome annotation.

### The western pleasure subpopulation

In the western pleasure subpopulation, candidate genes located on ECA7 are also of interest when considering selection for the phenotype exhibited by individuals from this group. Western pleasure is a performance style in which horses need to show a relaxed and collected disposition during competition. In this event, animals are asked by their riders to walk, trot and lope in both directions of the arena, as well as to gently come to a stop and to stand still at command. In order to excel in western pleasure, individuals need to have a calm and quiet temperament and smooth, controlled movements. Moreover, elite athletes are expected to possess cognitive and decision-making skills that are not exclusively dependent on the rider in order to excel in competitions. Genomic selection analyses show a collection of 24 genes located within ROI on ECA7 (see Supplementary Table 13). Interestingly, half (12) of these ECA7 genes are associated with cognition, behavior and nervous system phenotypes, including *AARS, GRIK4, NTM, PGLS*, and *SORL1*.

### The working cow subpopulation

Working cow horses, also known as reined cow horses, perform in events in which they are asked by the rider to work a single cow in an arena, performing specific maneuvers that include circling the cow, directing it in specific patterns, and performing a reining routine. This sport is similar to cutting because horses are also required to work alongside a cow, and for that they must possess “cow sense.” Genes associated with these phenotypes were identified within genomic signatures of selection in the working cow subpopulation, such as *DRD2* (ECA7:21.8 Mb) and *GRID2* (ECA3:44.7 Mb), associated with cognitive and behavioral traits in humans. The *APP* gene (ECA26:23.5 Mb), one of the top candidate genes for central nervous system and skeletal muscle development in this subpopulation, is involved in neural growth and maturation during brain development, and has been associated with the pathology of Alzheimer's disease in humans (Coronel et al., 2018).

Across all the subpopulations, approximately 90% of significant *d*_*i*_ windows were also observed by hapQTL as ancestral haplotypes; further, all 6 ROI identified by hapFLK were also significant either by *d*_*i*_statistics or hapQTL analysis. These results corroborate the efficacy of the methods utilized in this study, and show that the use of a combination of different methods to detect genomic selection, coupled with hapQTL, increases the chances of confidently identifying a larger spectrum of regions undergoing selection. It is also interesting to note that the hapFLK analysis identified far fewer selection signatures in the present study than *d*_*i*_. hapFLK is expected to be more stringent than *d*_*i*_, which typically suffers from selection bias and false positives; this is because hapFLK accounts for haplotype structure of each subpopulation for the significance calculation (Fariello et al., 2013). Moreover, these statistical tests can capture different selection signals: hapFLK is not as powerful for detection of more ancient signatures of selection, whereas *d*_*i*_ may fail to detect signatures of selection when SNPs within a window are not in high LD with the causative variant (Kijas et al., 2012; Fariello et al., 2013). Therefore, only the top 0.1% of *d*_*i*_ windows was considered for this analysis, which is consistent with studies in other species using similar approaches (Kijas et al., 2012; McRae et al., 2014; Zhao et al., 2015; Purfield et al., 2017).

Limitations to our study include the fact that several of the significant ROI identified did not contain protein-coding genes annotated in the current equine genome assembly (EquCab2). We hypothesize that this is due to limitations in the current equine genome annotation for regulatory regions, non-coding RNAs and sequence motifs, which might be driving selection in some of the identified ROI but are not identified as such in EquCab2. However, there are 20,449 protein coding genes annotated in EquCab2, so pathway analysis still constitutes a useful tool to predict putative functionality of candidate genes within ROI. Another potential limitation to the approach used in this study is that population differentiation at the genome level is not always a product of selective sweeps; it can also arise due to inversions and random genetic drift, for example. This can be tested with functional studies, which are not part of the scope of this work. Other limitations include arbitrary significance thresholds utilized in different detection methods for genomic selection (which might hinder the ability to detect ROI just below said threshold), and the possibility of false positives stemming from each statistical method used. Also, the close genetic relatedness among the performance groups used in this study hinder the ability to detect signatures of selection due to the occurrence of a high number of shared ancestral haplotypes among subpopulations. These subpopulations are comprised of individuals belonging to the same breed, that have been selectively bred for only approximately 60 years; thus, a high level of genetic similarity between different subpopulations is to be expected. Finally, pathways analyzed for gene prioritization were limited to skeletal muscle development, CNS and metabolism, while selection may also be occurring for other less obvious phenotypes of interest for each subpopulation.

As shown by previous work (Petersen et al., 2013b), signatures of selection are evident in all major breeds of horse, and understanding the alleles and functional bases for their phenotypic effects will greatly expand our understanding of equine biology. The aforementioned work (Petersen et al., 2013b) utilized 744 individuals from 33 breeds, and a low-density 54K SNP genotyping array, to identify breed-specific signatures of selection using *d*_*i*_ statistics calculated in 500-kb windows. *d*_*i*_ windows falling into the 99th percentile of the empirical distribution (a total of 33 for each breed) were considered significant. Among the top 33 *d*_*i*_ windows identified for the Quarter Horse, 4 are shared with the top 218 *d*_*i*_hits in this study: ECA2:95.7 Mb (shared by the cutting, western pleasure and working cow subpopulations), ECA3:26.5 Mb (found in the western pleasure subpopulation), ECA4:19.4 Mb (shared by the racing and working cow subpopulations), and ECA18:65.2 Mb (found in the cutting group). While none of the significant signatures of selection identified by hapFLK in this study were found in the work performed by Petersen et al. (2013b), a total of 18 of the 33 top *d*_*i*_hits found in that study were also significant by hapQTL analysis in the present work, constituting shared ancestral haplotypes between individuals of one or more QH subpopulations. Among those, one of particular interest is the ROI located on ECA18:66.3 Mb which contains the myostatin (*MSTN*) gene, where a SINE insertion allele in the promoter region is associated with an increased gluteal muscle type 2A fiber proportion and contributes to the outstanding sprinting ability of this breed (Petersen et al., 2013b). A common 780.7 kb haplotype encompassing *MSTN* was shared by 91.3% of Quarter Horses used in the study (Petersen et al., 2013b), in agreement with the hapQTL results presented herein, where this genomic region constitutes an ancestral haplotype shared by all 6 QH subpopulations (Supplementary Table 3). The use of 2 million SNP markers and the comparison of subpopulations within the QH breed, as opposed to comparing the breed as a whole to other horse breeds, likely contribute to differences between the results of this study and that of Petersen et al. (2013b).

Our work represents a more thorough study of selection in horse breeding by leveraging high-density SNP data within a single breed to demonstrate that there are many regions of selection ongoing within each of the performance categories of the American Quarter Horse. Moreover, the use of a combination of different statistical and haplotype analysis methods in this study has allowed for a broader range of detection of selection signatures. Although at present we cannot assess issues of false positives, and gene missingness due to incorrect/incomplete genome annotation, we consider this an excellent start. We expect that future high-density analyses will be forthcoming across racing breeds and again across a wide diversity of other breeds, and that from this collection key haplotypes and functional alleles underlying selection will be discovered and used to further select and hopefully improve the populations.

## Author contributions

FA: performed the signatures of selection, candidate gene, and pathway analyses; RS: managed the data pipeline and performed the imputation; MM, FA, and JM: designed the study; FA, JM, and MM: wrote the manuscript; RS: edited the manuscript. All authors have read and approved the final version.

### Conflict of interest statement

The authors declare that the research was conducted in the absence of any commercial or financial relationships that could be construed as a potential conflict of interest.

## Footnotes

^1^Available online at https://www.aqha.com/media/17914/2016-annual-report.pdf. (Accessed 21 January 2018).

^2^Available online at https://www.aqha.com/media/17917/2015-annual-report.pdf. (Accessed 21 January 2018).

^3^animalgenome.org. https://www.animalgenome.org/repository/horse/.

## Supplementary material

Supplementary Figures 1–6 Circos plots showing genome-wide di values (red layer), hapQTL values (orange layer), and hapFLK values (blue layer) across all 31 autosomes for all 6 QH subpopulations.

Supplementary Table 1 List of seed genes obtained using the PolySearch 2.0 algorithm based on skeletal muscle development, central nervous system development and metabolism.

Supplementary Table 2 Significant di windows in all 6 QH subpopulations.

Supplementary Table 3 Significant hapQTL windows in all 6 QH subpopulations.

Supplementary Table 4 Unprioritized list of genes retrieved for all 6 QH subpopulations.

Supplementary Table 5 Genes identified by Endeavor using the skeletal muscle seed gene list in all 6 QH subpopulations ranked by p-value. Each column on the table corresponds to a human-based data source used by Endeavor-GW to rank the provided list of genes based on the seed genes. These are: gene and protein function, chemical information, biomolecular pathways, phenotypic information, interaction networks, expression profiles, expression ontologies, and sequence-based features.

Supplementary Table 6 Genes identified by Endeavor using the central nervous system seed gene list in all 6 QH subpopulations ranked by p-value. Each column on the table corresponds to a human-based data source used by Endeavor-GW to rank the provided list of genes based on the seed genes. These are: gene and protein function, chemical information, biomolecular pathways, phenotypic information, interaction networks, expression profiles, expression ontologies, and sequence-based features.

Supplementary Table 7 Genes identified by Endeavor using the metabolism seed gene list in all 6 QH subpopulations ranked by p-value. Each column on the table corresponds to a human-based data source used by Endeavor-GW to rank the provided list of genes based on the seed genes. These are: gene and protein function, chemical information, biomolecular pathways, phenotypic information, interaction networks, expression profiles, expression ontologies, and sequence-based features.

Supplementary Table 8 Examples of candidate genes for phenotypic traits of interest with their respective functions for each QH subpopulation.

Supplementary Table 9 FunRich3.0 functional enrichment and pathway analysis results for the cutting QH subpopulation.

Supplementary Table 10 FunRich3.0 functional enrichment and pathway analysis results for the halter QH subpopulation.

Supplementary Table 11 FunRich3.0 functional enrichment and pathway analysis results for the racing QH subpopulation.

Supplementary Table 12 FunRich3.0 functional enrichment and pathway analysis results for the reining QH subpopulation.

Supplementary Table 13 FunRich3.0 functional enrichment and pathway analysis results for the western pleasure QH subpopulation.

Supplementary Table 14 ROI identified by both the current and previous studies.

Supplementary Table 15 FunRich3.0 functional enrichment and pathway analysis results for the working cow QH subpopulation.
