# Supplementary figures and images for: Genome-Wide Signatures of Selection Reveal Genes Associated With Performance in American Quarter Horse Subpopulations
Source: Front Genet. 2018 Jul 19;9:249. doi: 10.3389/fgene.2018.00249 (PMC6060370; doi:10.3389/fgene.2018.00249)

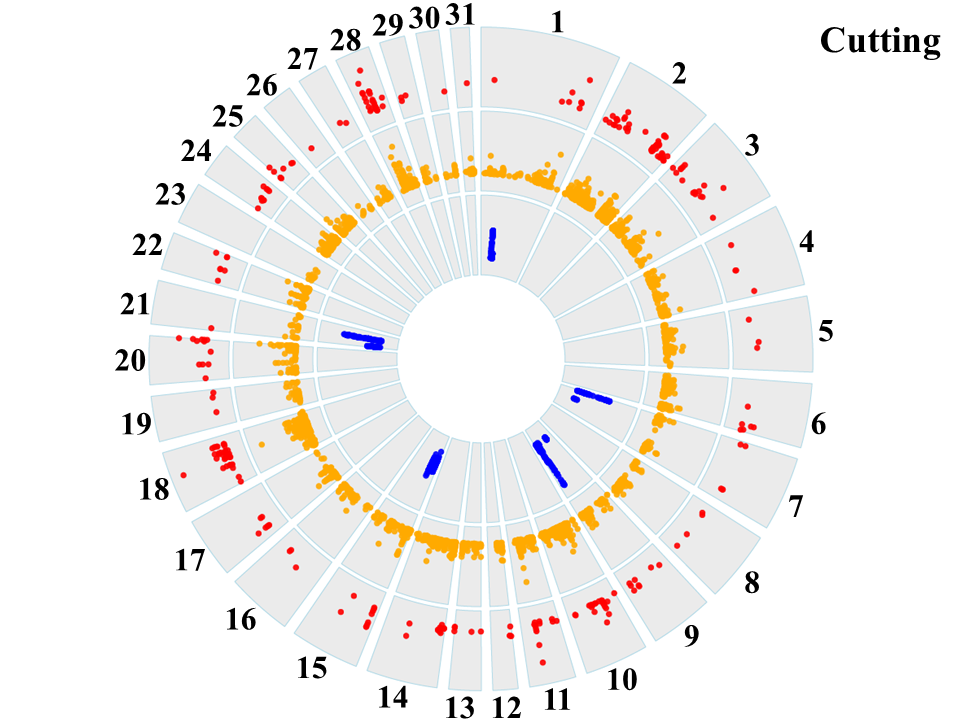

Supplement: Supplementary file 1 [file Image_1.TIF]

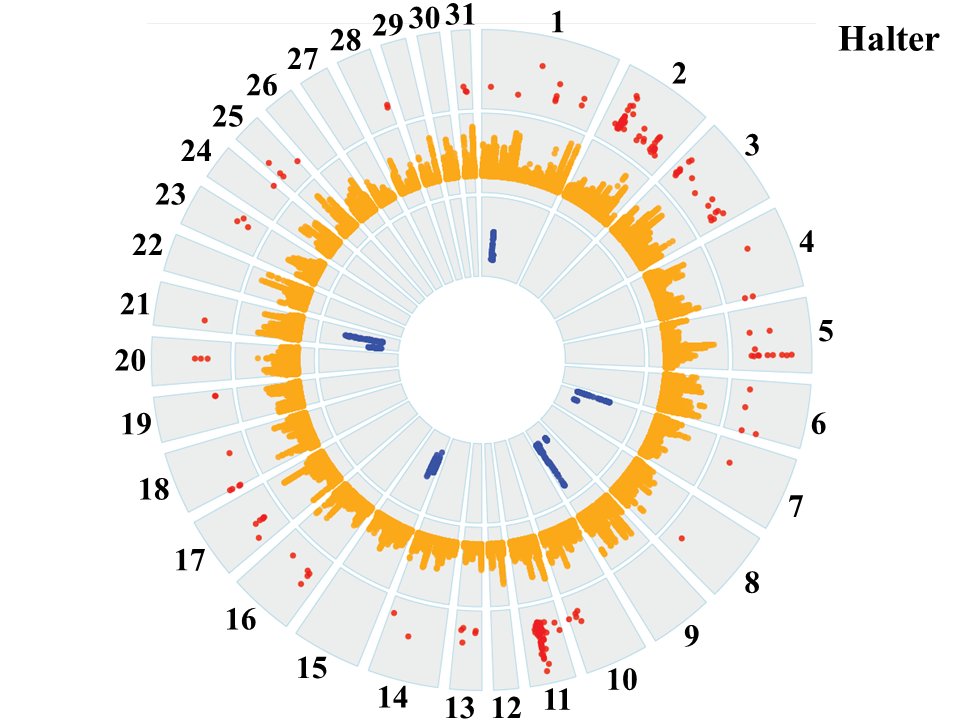

Supplement: Supplementary file 2 [file Image_2.TIF]

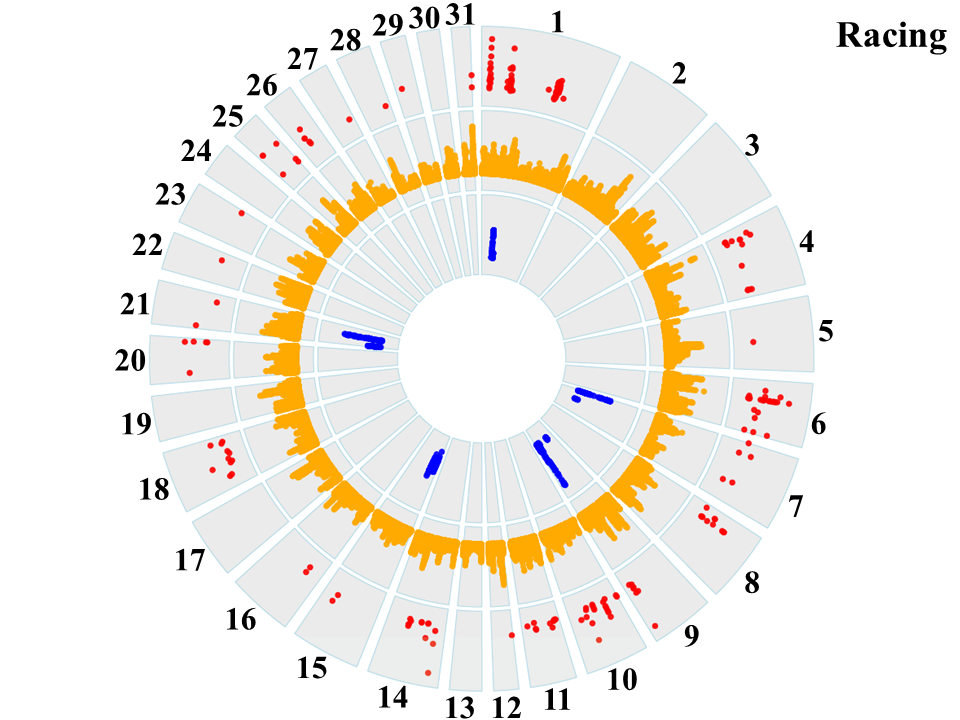

Supplement: Supplementary file 3 [file Image_3.TIF]

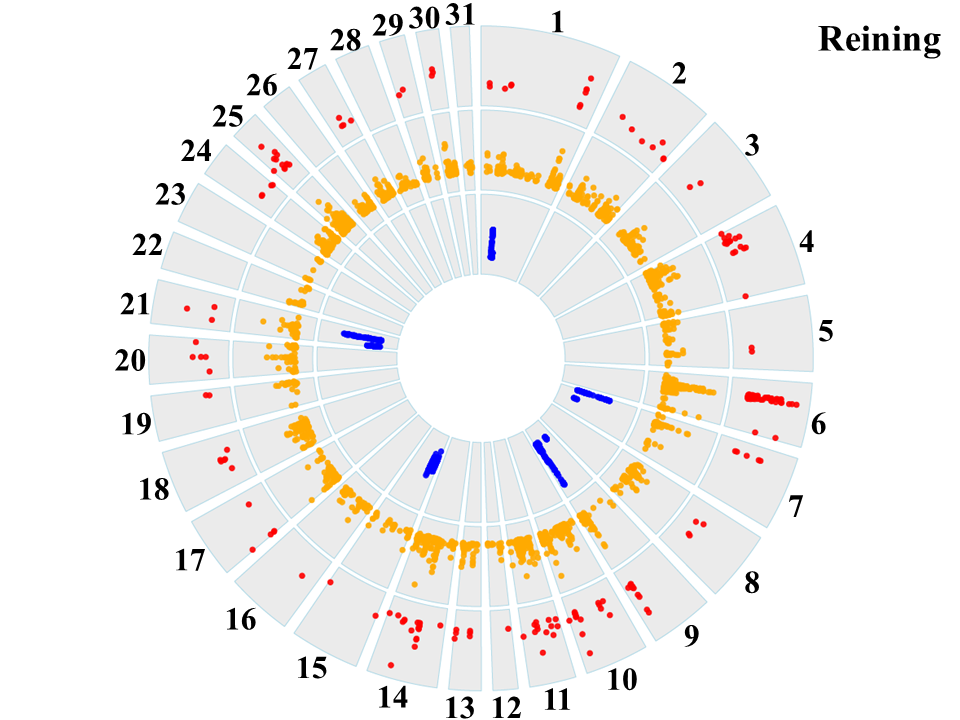

Supplement: Supplementary file 4 [file Image_4.TIF]

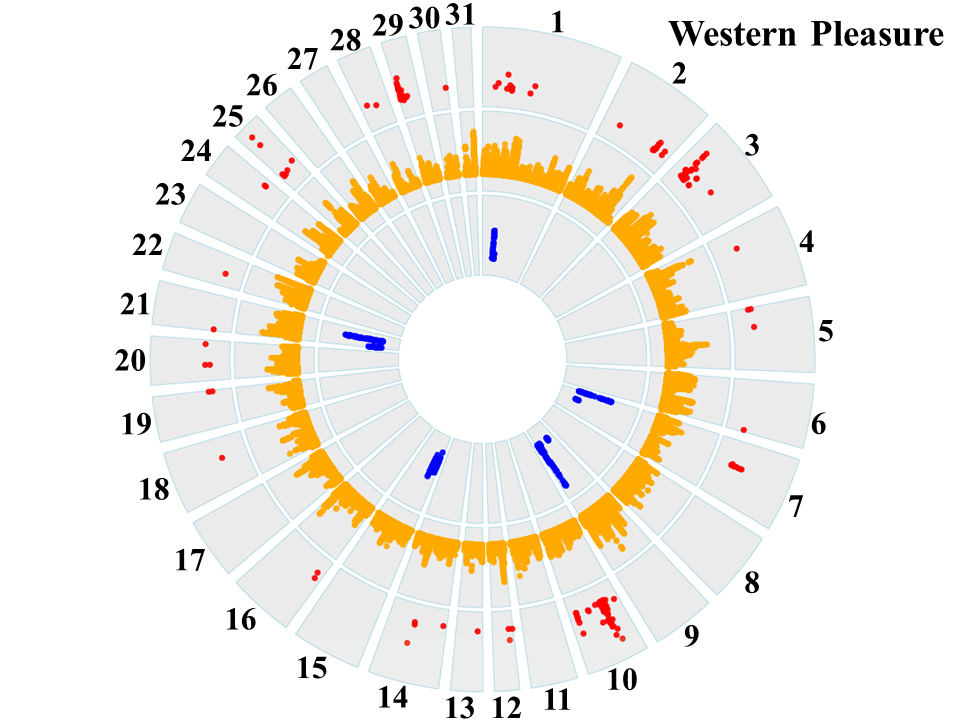

Supplement: Supplementary file 5 [file Image_5.TIF]

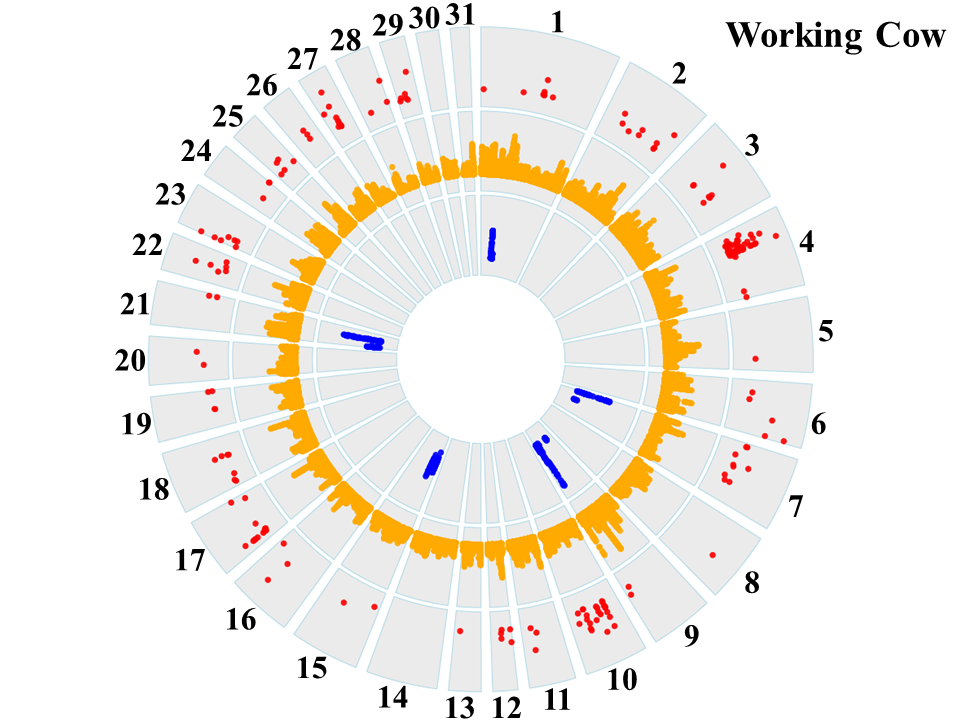

Supplement: Supplementary Figures 1–6 — Circos plots showing genome-wide di values (red layer), hapQTL values (orange layer), and hapFLK values (blue layer) across all 31 autosomes for all 6 QH subpopulations. [file Image_6.TIF]
