# Supplementary material for: Genome-Wide Signatures of Selection Reveal Genes Associated With Performance in American Quarter Horse Subpopulations
Source: Front Genet. 2018 Jul 19;9:249. doi: 10.3389/fgene.2018.00249 (PMC6060370; doi:10.3389/fgene.2018.00249)
Supplement: Supplementary Table 8 — Examples of candidate genes for phenotypic traits of interest with their respective functions for each QH subpopulation. [file Table_8.DOCX]

| **Supplementary Table 8.** Examples of candidate genes for phenotypic traits of interest with their respective functions for each QH subpopulation. | | | | |
| --- | --- | --- | --- | --- |
| **QH Subpopulation** | **Characteristics** | **Candidate Gene** | **Gene Function** |  |
| Cutting | Horse must keep a cow away from the herd, showing cow sense, attentiveness and courage. | *GAD1* | Central nervous system development |  |
|  |  | *ERBB3* | Metabolism |  |
|  |  | *NEB* | Skeletal and cardiac muscle function |  |
| Halter | Horse must have a good conformation, including degree of muscling, balance and structural correctness. | *CCL2* | Metabolism |  |
|  |  | *ALCAM* | Central nervous system development |  |
|  |  | *MYH11* | Skeletal muscle development |  |
| Racing | Horse must have athleticism, speed and stamina. | *THSD4* | Heart and lung function |  |
|  |  | *BAG3* | Skeletal muscle development |  |
|  |  | *HSPD1* | Behavior and cognition |  |
| Reining | Horse must be attentive in order to execute commands and specific maneuvers. | *ITGB2* | Skeletal and cardiac muscle function |  |
|  |  | *IGFBP1* | Glucose metabolism |  |
|  |  | *BACE1* | Cognition, behavior |  |
| Western Pleasure | Horse must be calm, quiet and attentive in order to execute commands. | *SLC27A1* | Metabolism |  |
|  |  | *GRIK2* | Neurological function |  |
|  |  | *MYO6* | Skeletal muscle function |  |
| Working Cow | Horse must have cow sense, smoothness of movement and reining ability. | *APP* | Neurological function |  |
|  |  | *DRD2* | Memory, learning |  |
|  |  | *SLC1A2* | Metabolism |  |
